# Sjogren's Syndrome and TAM Receptors: A Possible Contribution to Disease Onset

**DOI:** 10.1155/2019/4813795

**Published:** 2019-05-13

**Authors:** Richard Witas, Ammon B. Peck, Julian L. Ambrus, Cuong Q. Nguyen

**Affiliations:** ^1^Department of Oral Biology, College of Dentistry, University of Florida, Gainesville, FL, USA; ^2^Department of Infectious Diseases and Immunology, College of Veterinary Medicine, University of Florida, Gainesville, FL, USA; ^3^Division of Allergy, Immunology and Rheumatology, SUNY at Buffalo School of Medicine, Buffalo, NY, USA; ^4^Center for Orphan Autoimmune Diseases, University of Florida, Gainesville, FL, USA

## Abstract

Sjogren's syndrome (SS) is a chronic, progressive autoimmune disease featuring both organ-specific and systemic manifestations, the most frequent being dry mouth and dry eyes resulting from lymphocytic infiltration into the salivary and lacrimal glands. Like the related autoimmune disease systemic lupus erythematosus (SLE), SS patients and mouse models display accumulation of apoptotic cells and a Type I interferon (IFN) signature. Receptor tyrosine kinases (RTKs) of the Tyro3, Axl, and Mer (TAM) family are present on the surface of macrophages and dendritic cells and participate in phagocytosis of apoptotic cells (efferocytosis) and inhibition of Type I IFN signaling. This review examines the relationship between TAM receptor dysfunction and SS and explores the potential contributions of TAM defects on macrophages to SS development.

## 1. Overview of Sjogren's Syndrome

Sjogren's syndrome (SS) is an autoimmune disorder characterized by a dysfunction of the salivary and lacrimal glands that can be associated with various systemic manifestations and other autoimmune diseases, such as rheumatoid arthritis (RA) and systemic lupus erythematosus (SLE). It is considered the second most common autoimmune disease, after rheumatoid arthritis. Its prevalence is estimated at 1% (0.1–4.8%) with an incidence of 7 per 100,000 in the United States. It is estimated that roughly 4 million Americans have SS with 90% of them being women and 50% of them having SS in association with another autoimmune disease [1–3]. The incidence of SS is found to be lower in China and higher in Japan [4]. There is a great deal of clinical. variability such that some patients may only have dry eyes and/or dry mouth, while others may have systemic manifestations including lung disease, kidney disease, and lymphoma. The 2016 American College of Rheumatology (ACR)–European League Against Rheumatism (EULAR) criteria for SS include symptoms of oral and/or ocular dryness or extra glandular manifestation along with object indicators including a minor salivary gland biopsy showing lymphocytic infiltration, anti-Ro antibodies, positive ocular staining score, reduced Schirmer's test, and/or reduced unstimulated salivary flow [5]. These criteria have undergone and will continue to undergo revision as more is learned about the disease and its protean manifestations.

Involvement of the eyes is one of the defining features of SS. In the United States, as many as 25% of the patients who present with dry eyes have SS. In China, one study estimated that only 1.9% of the dry eye patients had SS [6, 7]. The lack of lacrimal gland secretions can result in corneal ulceration and perforation, conjunctivitis, uveitis, scleritis and episcleritis, optic neuritis, and orbital inflammation all of which can be infectious and/or “autoimmune” [6]. Involvement of the salivary glands is the second defining feature of SS. Patients with SS experience dry mouth, burning sensation in their mouth, loss of sense of taste and smell, inability to eat, chew and swallow food, speaking difficulty, and weight loss. Complications of SS in the oral cavity include dental caries, gingivitis, dry and cracked lips, depapillation of the tongue, oral ulcers, and infections especially with fungi [8].

Many patients with SS will have other systemic manifestations. Lung involvement occurs in 9–75% of patients with SS [9, 10]. The most common lung finding in SS patients, which occurs in roughly 1% of patients, is lymphocytic interstitial pneumonia (LIP) [11]. Kidney involvement occurs in approximately 5% of patients with primary SS (pSS) [12–14]. The majority of SS patients have lymphocyte predominant tubulointerstitial nephritis (TIN) although some patients have glomerular disease. Almost all patients with SS will have some issues with the gastrointestinal tract. Besides dry mouth, patients can have difficulty swallowing and gastrointestinal dysmotility including gastroesophageal reflux, constipation, and diarrhea [15–18]. Peripheral nervous system manifestations occur in 16% of patients with SS and include pure sensory neuropathy, sensorimotor neuropathy, cranial nerve involvement, mononeuritis multiplex, and polyradiculoneuropathy [19, 20]. The involvement of the genitourinary tract is one of the most disabling manifestations of SS for women. Vaginal dryness has been identified in 53% of SS patients and is often associated with dyspareunia and sexual dysfunction [21, 22]. Musculoskeletal complaints occur in most patients with SS. The most common complaints are arthralgias without frank arthritis although synovitis can occur in 15-35% of patients [23, 24]. The most typical joint involvement is the knees and the small joints of the hands and wrists. Arthritis tends to be nondeforming and not associated with erosions.

The most life-threatening manifestation of SS is lymphoma, which occurs in 5-10% of patients [25, 26]. The lymphomas are generally non-Hodgkin's B cell lymphomas that may be various histological subtypes including follicular lymphoma (FL), large B cell lymphoma (LBCL), and marginal zone lymphoma (MCL) [27]. Interestingly, the tumors may start not only in the salivary glands but also in other mucosal lymphoid tissues, such as Peyer's patches.

## 2. Lessons Learned from the C57BL/6.NOD-*Aec1Aec2* Mouse Model of SS

The first murine model identified to naturally develop salivary and lacrimal gland dysfunction consistent with human SS was the NOD mouse [28]. While these mice develop both a type 1 diabetes (T1D) and SS-like disease, the two autoimmune diseases were shown to result from different genetic regulations. The T1D phenotype has a strong dependence on a single MHC haplotype, whereas the SS-like phenotype is far more permissive and a feature that has permitted separation of the two diseases. This was first demonstrated in the NOD.B10Sn-*H2^b^*/J mouse derived by replacing the MHC locus of the NOD mouse first with the *H-2b* MHC of the C57BL/6 strain [29], then later with the *H-2q* MHC [30]. These recombinant inbred mice do not develop T1D, but continue to develop SS-like disease characterized by lymphocytic infiltration of the salivary and lacrimal glands, as well as pulmonary disease, renal disease, and autoantibodies [31, 32]. C57BL/6.NOD-*Aec1Aec2* was generated by breeding the combination of insulin-dependent diabetes (Idd) susceptibility interval 3 and 5 loci derived from the NOD mouse strain on the C57BL/6 background, which fully recapitulated the SjS phenotype [33, 34].

Based on an extensive published literature describing the pathology and accompanying histology of SS in human patients and SS-like disease in mouse models, there is a strong consensus that SS is a systemic autoimmune disease. However, like most autoimmune diseases, the causative agents and apparent dysregulated immune responses remain an unresolved mystery. As discussed, SS patients present in clinics with a wide range of symptoms and usually years after onset only confound diagnosis and potential for research into the various underlying etiologies. Nevertheless, the presence of autoantibodies, macrophages, T and B lymphocytes, and natural killer (NK) cells within the salivary and lacrimal glands of CD57BL/6.NOD-*Aec1Aec2* mice at the time of dysfunction supports the concept that an adaptive immune response is a major feature, particularly in the later stages of disease. This concept is strongly supported by the molecular studies by Delaleu et al. [35, 36] in the C57BL/6.NOD-*Aec1Aec2* mouse model indicating the presence of a classical MHC-dependent, T and B cell-mediated immune response. Interestingly, these molecular studies also indicate participation of mast cells, an intriguing finding that thus far has been ignored.

Autoimmunity is generally simplified as an interaction between an inducing environmental trigger and a host's genetic predisposition. Attempts to identify genetic factors that impose a predisposition to specific autoimmune diseases when the environmental triggers remain undefined represent a herculean task. Since individual autoimmune diseases, e.g., ankylosing spondylitis and T1D, have been shown to associate well with specific MHC haplotypes, it is assumed that SS disease susceptibility will also have an association with specific MHC haplotypes as well. Unfortunately, the underlying molecular, biological, and cellular processes involved in progression from a normal immune response to an apparently uncontrolled autoimmune response, revealed by the appearance of the covert clinical disease, remain poorly defined. Mutations and/or altered activities within any element involved in these response processes may affect downstream signaling even after normal antigen recognition by MHC molecules. Although data from the *Sjögren Big Data Project* [37] are beginning to identify MHC haplotypes associated with a predisposition for SS, the wide range of haplotypes being observed suggests a permissive association or the existence of multidisease subtypes or both. Again, data emerging from the various mouse models of SS are consistent with this concept.

The fact that SS, like SLE and other rheumatoid diseases, has been marked as disease with a strong Type I interferon (IFN) signature suggests a possible viral etiology. However, support for this possibility is complicated by the fact that the various viral diseases examined thus far are highly prevalent in normal human populations. Viruses studied include hepatitis B, hepatitis C, human T-cell leukemia virus type 1 (HTLV-1), mumps, and cytomegalovirus (CMV), but none of these have received widespread support as an environmental trigger. On the other hand, in-depth molecular analyses of genes and signaling pathways activated during the early inflammatory stage of SS-like disease development in C57BL/6.NOD-*Aec1Aec2* mice are consistent with an immune response towards a dsRNA virus, possibly of the *Picornaviridae* family (e.g., coxsackie, encephalomyocarditis, and rhinoviruses) or the *Reoviridae* family (e.g., rotovirus) [38]. Support for this conclusion rests in three distinct, yet interactive, observations. First, the three PRRs activated in the innate phase of disease (i.e., Tlr3, Tlr4, and Mda-5) are receptors involved in the downstream activation of the IFN-based response against dsRNA viruses. Second, genes associated with the various innate cell-autonomous immune effector mechanisms exhibit upregulated expressions totally consistent with an anticytoplasmic viral response. Third, expressions of Trim and Socs molecules that regulate IFN promote an activation, not a downregulation, of innate immunity. While we currently favor this viral etiology hypothesis in the C57BL/6.NOD-*Aec1Aec2* mouse model, whether these data are translatable to human SS remains unknown.

### 2.1. Stages of SS Development

**Figure 1:**
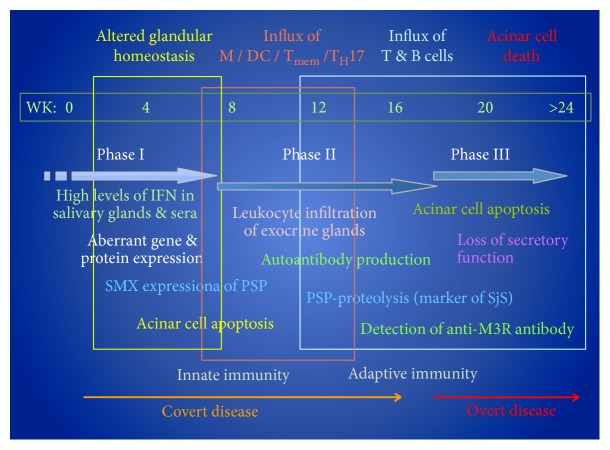
The temporal development and onset of pSS-like disease and pathology of the C57BL/6.NOD-*Aec1Aec2* mouse model. During Phase I (0-8 weeks), increased acinar cell apoptosis is detected along with elevated IFN signaling. Phase II (8-16 weeks) is characterized by an innate immune response and lymphocytic infiltration into the exocrine glands. Phase III (over 16 weeks) features an adaptive immune response with production of M3R autoantibodies and measurable loss in exocrine function. M: macrophage; T_mem_: memory T cells; T_H_17: T helper 17 cells; pSjS: primary Sjogren's syndrome; DC: dendritic cell; IFN: interferon; PSP: parotid secretory protein; SMX: submandibular gland; M3R: muscarinic type 3 receptor.

One of the attractive features of mouse models in the study of human disease is the ability to manipulate both the environment and the genetics of the test animals. Temporal studies of disease in animal models permit detailed investigations into what changes are occurring in both molecular and cellular processes during initiation, development, and subsequent onset of the disease. Temporal genome-wide microarray studies of the C57BL/6.NOD-*Aec1Aec2* mice from predisease to a full overt clinical SS-like disease have revealed that large numbers of molecular processes are either activated or downregulated and these processes are in constant flux [36]. Most importantly, these changing processes correspond to the advancing pathology observed in the salivary and lacrimal glands, as well as lung and kidney tissues, and identify heretofore unknown bioprocesses involved in disease development that have not been known in human SS patients. Extensive studies into its pathology have permitted graphing the temporal progression of disease, including the elements of the immunological attack against the salivary and lacrimal glands. This process is presented in Figure 1.

The procedure of combining the developing pathology in the exocrine glands with differential gene expression profiles that identify gene sets defining functional cellular processes permits the ability to compare the cellular pathology versus molecular events. This procedure has shown that multiple disease susceptibility loci-dependent aberrations are occurring in salivary and lacrimal gland integrity and subsequent homeostasis prior to onset of detectable disease. These changes in glandular integrity, including increased cellular apoptosis, occur just ahead of the inflammatory and innate responses characterized by the definable Type I IFN signature. This phase of covert disease is predominantly dependent on genes located outside of the SS-predisposing *Aec1* and *Aec2* loci [36]. However, following a quiescent phase of transcriptional stability, a new set of genes that clearly identifies the clinical onset of an active SS disease emerges exhibiting a relatively sudden and sustained upregulation. This gene set defines T-, B-, and NK cell-specific signal transduction pathways, alterations in lymphoid cell-associated focal adhesions, and cell-cell junctions, as well as the loss of neurotransmitter receptor activities [35, 36]. Overall, these pathology profiles verify the molecular profile and *vice versa*, while at the same time, indicating a complexity beyond a simple adaptive immune response.

## 3. The Role of Tyro3, Axl, and Mer Receptor Tyrosine Kinases

Tyro3, Axl, and Mer make up the TAM family of receptor tyrosine kinases. Like other receptor tyrosine kinases, TAMs receive an extracellular signal and respond by inducing autophosphorylation of tyrosine residues, recruiting downstream signaling molecules, and initiating intracellular transcriptional changes. TAM receptors are attracting increasing research interest due to their potential involvement in autoimmunity, cancer, and facilitation of viral infection through apoptotic mimicry [39–44]. TAM receptors are related through both sequence and functional homologies. Each member possesses two extracellular immunoglobulin-like domains at the amino terminus, two fibronectin type III domains, a hydrophobic transmembrane domain, and an intracellular tyrosine kinase domain at the carboxy terminus [45–47]. The human TAM receptors share 31-36% of their amino acid sequences within the extracellular portions and 54-59% homology within the intracellular tyrosine kinase domain [48]. While protein sizes of 97, 98, and 110 kilodaltons were expected for human Tyro3, Axl, and Mer, respectively, proteins of 100-140 for Axl and 165-205 for Mer were actually detected, as a result of posttranslational modifications to these proteins [46, 49–51]. A wide variety of outcomes can result from activation of TAM receptors. TAM receptor signaling has been implicated in regulation of inflammatory cytokine release, apoptotic cell phagocytosis (efferocytosis), cell proliferation and survival, and platelet stabilization [51–53].

### 3.1. TAM Receptor Interaction with Gas6 and Pros1

Growth arrest-specific protein 6 (Gas6) and protein S (Pros1) are well characterized TAM ligands. Both ligands are approximately 80 kilodaltons in size and are about 40% identical to one another at the protein level [54–56]. Structurally, Gas6 and Pros1 possess two laminin domains making up the carboxy terminal sex hormone-binding globulin domain (SHBG). The laminin domain binds to the immunoglobulin domain of the TAM receptor, causing dimerization and activation of the receptor. The Gla domains exist at the amino terminus of Gas6 and Pros1, and four epidermal growth factor-related domains (EGF) are present between the Gla and laminin domains. The Gla domains are characterized by a dense concentration of glutamic acid residues. These glutamic acid residues are posttranslationally modified into gamma carboxy glutamic acid (Gla) by gamma glutamyl carboxylase in a vitamin K-dependent reaction [57–59]. Ca^2+^ ions bind the Gla domains, facilitating folding, enhancing stability, and permitting binding to phosphatidylserine (PtdSer) [60].

Gas6 and Pros1 act as bridging molecules between TAM receptors and PtdSer exposed on the surface of apoptotic cells. Upon binding the immunoglobulin-like domain, Gas6 and Pros1 cause dimerization of TAM receptors and autophosphorylation of tyrosine residues within the tyrosine kinase domain, thereby recruiting additional molecules for downstream signaling. It has been observed that all three TAM receptors are activated by Gas6; however, Axl cannot be activated by Pros1 [61]. Additionally, while Mer and Tyro3 show limited activation in response to Gas6 without PtdSer, Axl absolutely requires both Gas6 and PtsSer for phagocytic activity within the retina and testes of mice [61].

### 3.2. TAM Receptor Expression

The TAM Gas6/Protein S system is an evolutionarily recent development found in vertebrates and prevertebrate chordates but lacking in sea urchin and *Drosophila* [62]. All 3 TAM receptors have been discovered in vertebrate embryonic tissue [25–27]. However, TAM knockout (KO) mice, including triple knockouts, are viable at birth and can survive for up to a year, suggesting that TAM receptor activity is not necessary during embryonic development [12]. TAM receptors are expressed nearly ubiquitously and have been discovered in tissues as diverse as the retinal pigmented epithelium (RPE) of the eye, Sertoli cells of the male reproductive system, platelets, and cells of the vascular and nervous system [40, 63]. This diversity in tissue expression is alluded to the origin of the name for the gene so-called *mer*, because mRNA for this gene was discovered in monocytes, epithelial cells, and reproductive cells [64]. Despite expression in an abundant array of tissues, much of the interest on TAMs has been focused on their role within the phagocytes of the immune system. Mer in particular is closely associated with macrophages. Mer has been determined to be a core macrophage antigen and a useful marker to distinguish macrophages from dendritic cells in both humans and mice [65, 66]. Axl is found on both macrophages and dendritic cells (DC) [67]. Whereas Tyro3 is expressed at low levels in these cells but is more highly expressed within the cells of the nervous system [68].

### 3.3. TAM Receptor Signaling in Efferocytosis

Efferocytosis is a critically important process for normal tissue maintenance. It has been estimated that approximately 150 billion cells (0.4% of the cellular mass of the human body) are turned over daily [69, 70]. Failure to properly remove apoptotic cells creates dire consequences for tissue function. The critical role for TAMs in efferocytosis was observed in several experiments involving TAM-deficient mice. Mer-deficient mice were found to develop blindness due to death of the photoreceptors (PR) in the retina. Mer was observed to be critical for daily pruning of the distal membrane segments of PRs by RPEs. Failure to perform this limited form of apoptotic engulfment leads to apoptosis of the PRs, retinal degeneration, and eventual blindness [71–74]. A similar phenomenon was observed within the testes of TAM-deficient mice. Normally, TAM-expressing Sertoli cells remove apoptotic cells generated within the testes. Sertoli cells from TAM-deficient mice have no ability to clear the apoptotic germ cells generated during meiosis, triggering death of germ cells and infertility [50].

Efferocytosis in other tissues of the body is largely carried out by phagocytes of myeloid origin. Macrophages primarily rely on Mer for phagocytosis of apoptotic cells, but the absence of Axl and/or Tyro3 also impairs efferocytosis [75]. In contrast, Axl and Tyro3 are critical for DC-mediated efferocytosis [75]. The mechanism of TAM contributions to efferocytosis has been characterized for Mer. Mer binds Gas6 or Pros1 bound to PtdSer on the surface of an apoptotic cell, Mer dimerizes, and autophosphorylates tyrosine residues within its tyrosine kinase domain. Two different mechanistic variations have been proposed where either Mer phosphorylates Vav1 leading to interaction with Rac1 and cdc42 [76] or Mer acts through Src and FAK also allowing Rac1 activation but in an *α*V*β*5 integrin-dependent manner [77]. In both models, Rac1 activation enables reorganization of the actin cytoskeleton to facilitate phagocytic engulfment of the apoptotic cell.

### 3.4. TAM Receptor Signaling in Dampening the Interferon Response

TLRs are pattern recognition receptors expressed on the surface of sentinel cells of the immune system. Each variant of TLR recognizes a conserved molecular pattern associated with bacteria, viruses, or fungi. Activation of TLRs by their ligand causes receptor dimerization and interaction with signaling adaptors, leading to the activation of several possible signaling pathways and the production of proinflammatory cytokines including Type I IFNs [78]. Type I IFNs are pleiotropic cytokines that initiate an antiviral state by impacting many aspects of the immune system. Type I IFNs facilitate antigen presentation by promoting DC maturation, migration, and cross presentation and boost both the cellular and humoral arms of the adaptive immune system [79]. Like many other cytokines, transcriptional changes brought about by Type I IFN signaling occur though Janus kinase/signal transducers and activators of transcription (JAK/STAT) signaling. Type I IFN binds the Type I IFN receptor (IFNAR) causing receptor dimerization and subsequent phosphorylation through JAKs. STATs bind the phosphorylated receptor and are then themselves phosphorylated by JAKs. The phosphorylated STATs dimerize, enter the nucleus, and initiate transcription of interferon-stimulated genes (ISGs) [79, 80]. TLR activation and Type I IFN signaling constitute important early steps in mobilizing the immune response against pathogen invasion.

Due to their powerful inflammatory effects, TLR signaling must be tightly regulated to avoid host damage. TAM receptors have been discovered to be critical regulators of TLR signaling as evidenced by TAM-deficient mice which develop profound autoimmunity and hyperresponsiveness to TLR ligands [81]. Regulation of TLR signaling has been best characterized for Axl in DCs [82]. Axl is normally expressed at low levels and induced in response to TLR3, TLR4, and TLR9 activation [82]. Triggering these TLRs stimulates the release of Type I IFN, and *Axl* is one of the many genes upregulated in response to this cytokine. Axl forms a physical complex with IFNAR, enabling the expression of the negative-regulatory SOCS1 and SOCS3 proteins [82]. The specific events permitting this shift from proinflammatory to immunosuppressive signaling of IFNAR have yet to be described.

**Figure 2:**
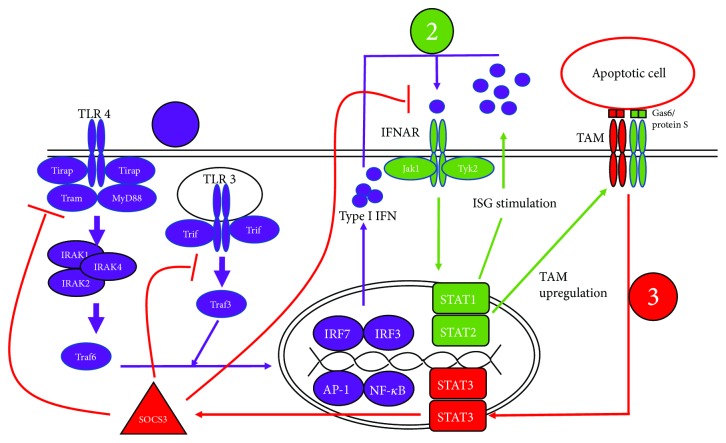
TLR and TAM receptor pathways in SS (1). Activation of TLR3 elicits the production of Type I IFN through the following transcription factors: interferon regulatory factors (IRF) 3/7, NF*κ*B, and AP-1 (2). Type I IFN initiates Janus kinase/signal transducer and activator of transcription (JAK/STAT) signaling through the IFNAR, stimulating the expression of interferon-stimulated genes (ISGs) including TAM receptors (3). TAM receptors form a complex with IFNAR, resulting in the upregulation of SOCS3 through JAK/STAT signaling.

SOCS proteins are a family of 8 proteins (CIS-SOCS7) that are induced by signaling through cytokine receptors and negatively regulate the same cytokine signaling pathways that induced their expression, composing a classical negative feedback loop [83, 84]. SOCS-mediated regulation occurs through two main mechanisms. First, the C terminal SOCS Box allows SOCS proteins to function as E3 ubiquitin ligases, resulting in the ubiquitination and subsequent degradation of JAKs and cytokine receptors through the proteasome [83, 85–88]. Second, unlike other SOCS family members, SOCS1 and SOCS3 possess a kinase inhibitory region (KIR) capable of directly binding to JAKs. This interaction blocks the catalytic site of JAKs, thereby preventing phosphorylation of STATs and inhibiting cytokine signaling [89, 90]. SOCS1 and SOCS3 possess much weaker ubiquitin ligase activity than the other SOCS proteins and primarily act through the second mechanism [84, 91]. Both methods, degradation of signaling components and blocking catalytic activity of JAKs, produce a similar outcome which is the inhibition of specific cytokine signaling pathways. Therefore, TAMs act as a set of brakes on the innate immune response that activate only after the response has already begun.  Figure 2 summarizes SOCS3 induction through TAM receptors.

## 4. Apoptosis in Sjogren's Syndrome

Salivary gland dysfunction can be mediated by various biological and immunological factors. One of the compelling factors is the role of glandular apoptosis or programmed cell death (PCD) in the initiation of the disease. Earlier works in the field have shown that acinar epithelial cells in SS expressed Fas (TNFRSF6) and FasL (TNFSF6) and underwent Fas-mediated apoptosis [92]. DNA strand breaks were detected mostly in the ductal epithelium and less in acinar tissues of patient salivary glands [93]. Salivary epithelial cells are constantly exposed to various biological and environmental stimuli, and some of these stimuli might have a detrimental effect on the cells, as demonstrated by Manoussakis et al. in which polyI:C which mimics viral dsRNA were able to induce anoikis and apoptosis via TLR3 [94]. And TLR3-mediated apoptosis can be mitigated by activating the peroxisome-proliferator-activated receptor-*γ* (PPAR*γ*) which was downregulated in salivary glands of SS patients [95]. Okuma et al. have shown SS-like signs like dacryoadenitis and anti-SSA/SSB autoantibodies can develop when genetically knockout the transcriptional regulator I*κ*B-*ζ* and apoptosis of epithelial cells can be observed in the absence of infiltrating lymphocytes [96]. Similarly, using the NOD animal model, Humphreys-Beher et al.'s group determined that Fas was highly expressed in only lacrimal and salivary glands at mice at the diseased age and double-stranded DNA (dsDNA) breaks were identified only on the epithelial cells in the absence of B and T cell infiltrates [97]. The group further determined that matrix metalloproteinase (MMP) 9 was expressed in the parotid and submandibular glands, suggesting a rampant breakdown of epithelia which results in uncontrolled PCD [98]. We have shown that cleaved products of caspase-3 can be detected as early as 4 weeks of age in the submandibular glands of SjS-susceptible C57BL/6.NOD-*Aec1Aec2* mice [99]. Interestingly, this mouse model also exhibited sexual dimorphism in apoptosis by which males and females undergo the apoptotic cellular event differently. Female SjS mice developed profound salivary gland apoptosis as evidenced by dsDNA breaks and cleaved caspases-3. In contrast, diseased male mice were able to impede the severe progression of PCD [100]. The result suggests that female SjS mice might have an intrinsic defect in apoptotic clearance allowing for the spread of unregulated cellular cell death. The factors that lead to salivary PCD and defect in apoptotic clearance in SjS remain speculative. Further investigations are needed to address these important mechanistic issues.

### 4.1. TAM Receptors in Autoimmune Diseases

The inability to remove dead cells and defects in negatively regulating IFN signaling has implications in autoimmunity. Apoptotic cells that are not promptly removed via phagocytes can progress to secondary necrosis, resulting in the leakage of intracellular contents. The DNA and other self-antigens released by these dying cells can act as damage-associated molecular patterns (DAMPS) and trigger the TLRs of innate immunity [101–104]. The activated TLRs then generate an inflammatory immune response, creating an escalating cycle of damage to self-tissue, leading to autoimmunity. Alternatively and perhaps synergistically, TLRs activated in response to normal microbial stimuli in the absence of negative regulation can also present a danger to the host. The cytokines produced through the Type I IFN response can generate host damage through chronic inflammation, further contributing to autoimmunity [78, 80].

Some of the earliest functional studies of TAM receptors exposed the critical role of TAM receptors in regulating the immune response. TAM-deficient mice feature hyperproliferation of lymphocytes and systemic autoimmunity including production of autoantibodies and antibody deposition in kidney glomeruli [81]. In fact, TAM-deficient mice have been described as acquiring a SLE-like phenotype [40]. SLE and SS are both chronic autoimmune diseases with systemic inflammatory profiles. SLE and SS both primarily affect women and share many similarities such as the development of SSA/Ro60 and SSB/La-reactive autoantibodies; the two diseases can occur together in secondary SS [105–108]. Both diseases also display impaired phagocytic activity. High levels of soluble TAM receptors have been reported in the sera of SLE and juvenile onset SLE patients [109, 110]. Soluble TAM receptors have been observed to inhibit TAM-mediated phagocytosis [49]. Both of these findings have been hypothesized to contribute to the failure of efferocytosis and the subsequent presentation of self-antigens and contribution to autoimmunity.

Due to the similarities in disease profile, it has been hypothesized that a similar mechanism involving a failure to remove apoptotic cells may be involved in the development of autoimmunity in SS [111]. SS is also characterized by high levels of Type I IFN in peripheral blood and activation of IFN-stimulated genes, further suggesting a role for TAM signaling dysfunction in the onset of SS [112–114]. Interestingly, activation of TLR3 has been demonstrated to incur salivary gland hypofunction through Type I IFN and IL-6 signaling in C57BL/6 mice [115]. NOD.B10Sn-*H2^b^* mice lacking the TLR signaling adaptor MyD88 were protected from developing both local and systemic manifestations of SS [32]. There is limited information on the role of TAM and TAM ligand in SS compared to what is known in SLE; however, several studies have been reported. Reduced levels of TAM mRNAs were detected in peripheral blood mononuclear cells (PBMCs) of pSS patients, and increased levels of soluble Mer correlated with the incidence of SSA/SSB autoantibodies and disease severity, as indicated by the SS disease activity index (SSDAI) score [116]. TAM ligand expression in SS remains controversial, with one group reporting no differences in Gas6 and ProS expression between pSS and control patient PBMCs and plasma [116] and Chen et al. finding plasma Gas6 concentrations to be lower in pSS patients than controls in the plasma and labial salivary gland [117]. Using microarray analysis, we have shown that salivary glands of C57BL/6.NOD-*Aec1Aec2* mice expressed significantly lower levels of *Socs2* and *Socs3*. Furthemore, we have found that *Tyro3* was upregulated, whereas *Axl*, *Mer*, and *Gas6* were downregulated at the clinical disease stage in comparison to age- and sex-matched C57BL/6 mice [111]. These conflicting results concerning TAM ligand expression in SS are also reflected in SLE where one study reported higher levels of Gas6 and lower Pros1 in the plasma of SLE patients [118], while other groups have detected decreased Gas6 in the plasma of SLE patients [119], or little difference in plasma Gas6 and Pros1 between controls and SLE patients [120]. It has been observed that while SOCS3 is upregulated in PBMCs and labial salivary gland of pSS patients, negative regulation of cytokine signaling fails to occur [121], suggesting that SOCS3-mediated reductions in inflammatory signaling are defective in SS. Together, these results suggest that the crucial TAM activities of suppressing IFN signaling and efferocytosis may be impaired in SS.

### 4.2. TAMs and Macrophages in SS

If the aberrant efferocytosis and unrestrained IFN signaling observed in SS involve TAM receptor signaling, it might suggest that the phagocytes of the immune system are likely to be intimately involved in these processes within the disease. As crucial participants in both efferocytosis and the initiation, maintenance, and resolution of inflammatory signaling, macrophages may play an underappreciated role in development of SS [104, 122, 123]. Macrophages are known to be among the early infiltrates into the salivary glands of NOD mice, preceding the arrival of DCs and B and T lymphocytes [124]. Furthermore, macrophage infiltration has been positively correlated with disease progression [125]. Macrophages present in the submandibular gland of NOD/ShiLtJ mice have been shown to produce high levels of the B cell chemokine CXCL13, potentially contributing to the development of ectopic germinal centers within the salivary glands [126]. The proinflammatory phenotype of macrophages in SS has led to the interest in the IFN signature within monocytes from pSS patients. Monocytes from pSS patients exhibit a strong Type I IFN signature that correlates with markers of disease activity [127]. Additionally, expression of the ISG MxA was elevated enough in monocytes of pSS patients to serve as a biomarker for the activation of the systemic IFN response in SS [128]. Interestingly, monocytes collected from PBMCs provided by pSS patients were determined to have lower phagocytic activity than those taken from healthy controls [129], similar to a phenomenon observed in macrophages from SLE patients [130]. From these data, it seems apparent that macrophages are primarily proinflammatory in SS and are failing to adequately perform their TAM-associated roles in efferocytosis and resolution of IFN signaling. A recent paper has reported that regulatory T cells (Tregs) stimulate macrophage efferocytosis by production of IL-13 which in turn stimulates macrophages to release IL-10 which acts in an autocrine-paracrine manner to upregulate Vav1 and activate Rac1, enabling apoptotic cell engulfment [131]. While the role of Tregs in SS remains understudied and characterized by conflicting reports [132–134], the ability of SS macrophages to respond to IL-13 and IL-10 represents a new aspect of efferocytosis to be investigated in SS. Further investigation will need to be performed to explore the relationship of TAM receptors, phagocytes, and the onset of autoimmunity in SS.

## 5. Conclusion

**Figure 3:**
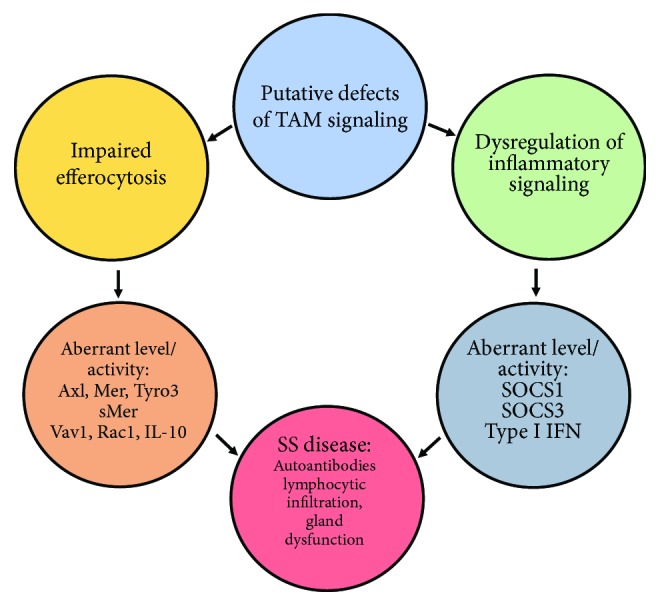
Summary of potential contributions of defective TAM signaling to SS. The etiology of SS is multifactorial, but it is known that innate immune dysfunction precedes adaptive immune dysfunction in the salivary glands. Here, we hypothesize that the TAM family of tyrosine kinases is involved in SS pathology through the TAM-mediated efferocytosis and Type I IFN regulatory pathways. We speculate that aberrations in TAM expression coupled with increased soluble Mer may account for the reported efferocytosis impairment, while downstream elements of efferocytosis signaling including Vav1 and Rac1 activation are unknown, as is SS macrophage response to IL-10 in the context of efferocytosis. Furthermore, we suggest that dysregulation of SOCS 1 and SOCS3 expression and activity may contribute to the overactive IFN signaling observed in SS. We postulate that these two failures in TAM signaling may be initial events in SS pathology that eventually lead to autoantibody generation, lymphocytic infiltration, and gland secretory dysfunction.

SS is a rheumatic disease with a well-characterized role for the adaptive immune system but a poorly understood etiology. Two aspects of SS that have gained significant attention in the last decade using human patients and animal models are the involvement of the Type I IFN system and unregulated glandular apoptosis. As discussed, TAM receptors are well-characterized molecules that contribute to both efferocytosis and dampening of the IFN response as illustrated in Figure 2. SOCS3 is an essential negative regulatory component in the proinflammatory process; however, its level and/or activity appeared to be significantly reduced in SS [111, 121]. While the status of SOCS3 expression in SS requires further clarification, lack of SOCS3 expression or SOCS3 activity represents an appealing mechanism to account for the failure to rein in inflammatory signaling in SS. Likewise, the observation that acinar epithelial cell apoptosis precedes lymphocytic infiltration and that SS macrophages exhibit reduced efferocytic activity presents another possible contribution of TAM dysfunction to SS through the accumulation of apoptotic debris and subsequent generation of a proinflammatory environment (unpublished data). As summarized in Figure 3, the two arms of TAM signaling appear to be disrupted in SS, potentially promoting the development of autoantibodies, lymphocytic infiltration, and overt disease. To fully understand the role of TAM receptors in SS, further studies will be needed to discern the innate response which involves in the activation of TLRs and the eventual upregulation of the TAM function. More importantly, additional studies will have to address the dysregulation of this complex process, specifically the role of SOCS proteins in the disease.
